# The effects of extracorporeal shockwave therapy on bone mineral density and microarchitecture in animal models of osteoporosis: a systematic review and meta-analysis

**DOI:** 10.3389/fresc.2025.1731044

**Published:** 2026-01-12

**Authors:** Xuelei Wen, Jiahao Xiangli, Qingzheng He, Zhanhai Yin

**Affiliations:** 1 Xi'an Physical Education University, School of Sports and Health Sciences, Xi'an, Shaanxi, China; 2 The First Affiliated Hospital of Xi'an Jiao Tong University, Department of Orthopedics, Xi'an, Shaanxi, China

**Keywords:** animal model, bone microarchitecture, bone mineral density, extracorporeal shockwave therapy, osteoporosis

## Abstract

**Objective:**

This systematic review and meta-analysis aimed to provide the first comprehensive, quantitative assessment of the efficacy of Extracorporeal Shockwave Therapy (ESWT) in improving bone mineral density (BMD) and key trabecular microarchitectural parameters (BV/TV, Tb.Sp, Tb.N, Tb.Th) in animal models of osteoporosis (OP).

**Methods:**

We systematically searched six electronic databases for relevant animal randomized controlled trials (RCTs). Data extraction and quality assessment were performed on the seven studies that met the inclusion criteria. All statistical analyses were conducted using RevMan 5.4.

**Results:**

The meta-analysis revealed that ESWT significantly improved all assessed parameters: (1) a significant increase in BMD [SMD = 2.12, 95% CI (1.50, 2.73), *P* < 0.00001] with low heterogeneity (*I*^2^ = 0%); (2) a significant increase in bone volume fraction [BV/TV, SMD = 2.26, 95% CI (0.20, 4.32), *P* = 0.03] with high heterogeneity (*I*^2^ = 76%); (3) a significant decrease in trabecular separation [Tb.Sp, SMD = −1.39, 95% CI (−2.64, −0.14), *P* = 0.03] with moderate heterogeneity (*I*^2^ = 63%); (4) a significant increase in trabecular number [Tb.N, SMD = 3.51, 95% CI (1.93, 5.10), *P* < 0.0001] with moderate heterogeneity (*I*^2^ = 62%); and (5) a significant increase in trabecular thickness [Tb.Th, MD = 0.09, 95% CI (0.04, 0.13), *P* = 0.0005] with low heterogeneity (*I*^2^ = 20%). Although initial heterogeneity for BV/TV, Tb.Sp, and Tb.N was high, sensitivity analysis identified the sources, and their removal resulted in more stable effect estimates.

**Conclusion:**

As the first quantitative synthesis in this field, this study provides preclinical evidence suggesting that ESWT is associated with improvements in bone density and optimizes bone microarchitecture. These findings establish a critical data foundation for ESWT as a potential non-invasive therapy for OP. However, it is important to note that the certainty of these findings is tempered by several factors, including the observed high heterogeneity for BV/TV, Tb.Sp, and Tb.N, the small total sample sizes across outcomes, and the potential for publication bias. The results are further constrained by exclusive reliance on animal models and a limited number of included studies. Future clinical research is required to validate these findings and further elucidate the underlying mechanisms.

## 1 Introduction

Osteoporosis represents a pervasive metabolic bone pathology affecting the entire skeleton, primarily driven by deficits in bone mineral content and erosion of microstructural connectivity. These core aberrations inevitably compromise bone's mechanical resilience, triggering a multifold increase in vulnerability to fragility fractures-particularly vertebral, hip, and distal radius fractures (1). Comprehensive meta-analyses document that osteoporosis currently afflicts 21.7% of the global adult cohort, constituting a critical public health challenge in aging societies (2), and the disease is associated with a high risk of fractures, particularly of the hip (3). As a global public health problem, OP and its resultant fragility fractures result in a severe impact on the quality of life for patients and place a substantial economic burden on society (4). Currently, clinical treatments primarily rely on anti-resorptive and anabolic agents (5, 6). However, long-term medication use can be associated with potential side effects, decreased patient compliance, and diminished therapeutic efficacy. Given these considerations, the exploration of safe, effective, and non-invasive physical therapies, such as Extracorporeal Shockwave Therapy (ESWT), as potential adjuncts to improve bone quality and enhance bone strength, represents a critical direction for investigation in this field.

ESWT is a non-invasive therapeutic modality, initially well-known for its successful application in urinary stone lithotripsy (7). In recent years, ESWT has also demonstrated considerable potential in the field of regenerative medicine (8–10). It utilizes high-energy acoustic pressure waves to induce a series of beneficial biological responses at the cellular and tissue levels through a mechanism of “mechano-biological effects”. Evidence suggests (11–13) that ESWT can effectively stimulate osteoblast proliferation and differentiation, promote angiogenesis, enhance levels of pivotal osteogenic factors including vascular endothelial growth factor (VEGF) and bone morphogenetic proteins (BMPs), and may suppress the overactivation of osteoclasts through osteoimmunological modulation. These mechanisms collectively suggest that shockwaves have the potential to reverse the bone metabolic imbalance induced by OP and to promote bone regeneration. However, it is also important to acknowledge that, like any therapeutic intervention, ESWT may carry potential dose-related harms or adverse effects, which warrant careful consideration in its application.

To accurately assess therapeutic efficacy, the quantitative analysis of bone microarchitecture is essential. Parameters such as bone mineral density (BMD), bone volume fraction/total volume (BV/TV), and microarchitectural indices including trabecular thickness (Tb.Th), trabecular number (Tb.N), and trabecular separation (Tb.Sp), serve as critical indicators for evaluating bone quality and predicting bone strength. In various animal models that simulate human OP, particularly postmenopausal OP (such as ovariectomized rodent models), numerous studies have explored the effects of ESWT intervention. These studies have generally reported positive trends, indicating that ESWT can, to a certain extent, improve bone density and microarchitecture in osteoporotic animals.

Although existing animal experiments have provided valuable evidence for ESWT in the context of OP, these studies exhibit significant differences in animal species, OP induction methods, shockwave energy parameters, treatment regimens, and intervention durations, leading to considerable heterogeneity in their findings. Furthermore, the limited sample size of individual studies restricts the robustness of their conclusions. To date, a systematic and quantitative synthesis of the overall effect size of ESWT on key microarchitectural indicators in OP is lacking in the literature. Therefore, responding to the critical gap in systematically integrated evidence, this study conducts the inaugural comprehensive and quantitative meta-synthesis of existing experimental data derived from osteoporosis (OP) animal models, utilizing a systematic review and meta-analytic framework. Our objective is to systematically evaluate the overall impact of ESWT on BMD, BV/TV, and key trabecular microarchitectural parameters (i.e., Tb.Sp, Tb.N, and Tb.Th). Through this research, we aim to **synthesize the existing preclinical findings** to assess the overall effect of ESWT on bone microarchitecture in animal models of OP. This quantitative analysis is intended to clarify the current state of the evidence, explore potential sources of inconsistency among studies, and provide a foundational summary that can help inform the rationale and design of future translational studies.

## 2 Data and methods

### 2.1 Inclusion and exclusion criteria

#### 2.1.1 Inclusion criteria

(1) Study Type: Randomized controlled *in vivo* animal experiments, limited to publications in English and Chinese. (2) Study Animals: Animal models of osteoporosis induced by any method (e.g., ovariectomy, glucocorticoid administration). (3) Interventions: The experimental group received Extracorporeal Shockwave Therapy (ESWT); the control group (e.g., sham surgery group, untreated group) received no ESWT or sham ESWT. (4) Outcome Measures: Studies that reported quantitative data for at least one bone microarchitectural parameter (e.g., BMD, BV/TV, Tb. Sp, Tb. N, Tb. Th).

#### 2.1.2 Exclusion criteria

(1) *in vitro* studies, reviews, human studies, conference abstracts, and letters. (2) Studies lacking a control group. (3) Studies that did not report any of the specified bone micro architectural parameters. (4) Studies where ESWT was combined with other bone-related treatments (e.g., drugs, growth factors) in a manner that precluded the isolation of ESWT's independent effects. (5) Duplicate publications and articles whose full texts were unobtainable.

### 2.2 Literature search strategy

#### 2.2.1 Search personnel

Two independent investigators executed the literature search following the predetermined protocol, maintaining complete autonomy throughout the process.

#### 2.2.2 Information sources and timeframe

To comprehensively identify relevant studies, this analysis involved a systematic search of multiple electronic databases, including PubMed, Scopus, Embase, the Cochrane Library, Web of Science, and the Chinese Biomedical Literature Database. The search encompassed all records from the inception of each database up to August 1, 2025. This study has been registered with PROSPERO (registration number CRD420251151338), and the registration details can be found at https://www.crd.york.ac.uk/PROSPERO/view/CRD420251178336.

#### 2.2.3 Search terms and execution

The search strategy was constructed by combining Medical Subject Headings (MeSH) with free-text words, covering terms such as “Extracorporeal Shockwave Therapy,” “Bone Mineral Content,” and “Bone Mineral Density.” These terms were combined using Boolean operators (e.g., “AND”, “OR”). The full detailed search strategy is presented in the Appendix. The literature search was performed by two independent researchers to ensure the rigor of the process and the objectivity of the results.

#### 2.2.4 Literature search strategy formulation

**Figure 1:**
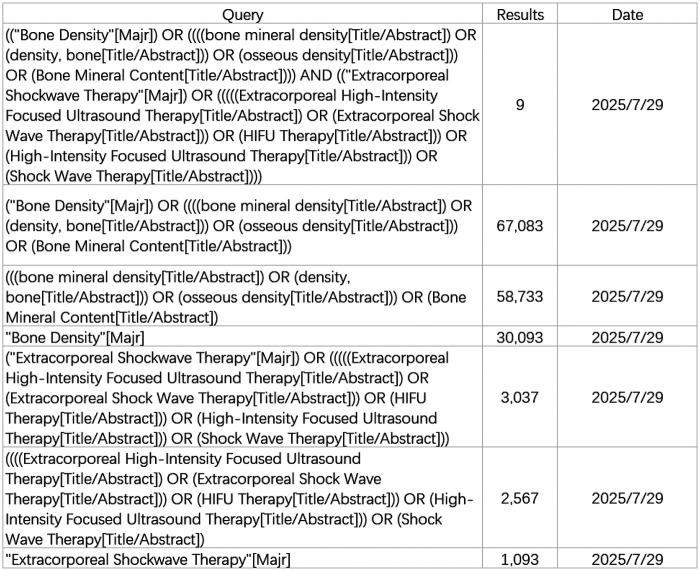
Pubmed database retrieval strategies.

Subject headings coupled with free-text keywords were jointly deployed in the retrieval process. The search strategies for PubMed is presented as examples in Figure 1.

### 2.3 Literature screening process

**Figure 2:**
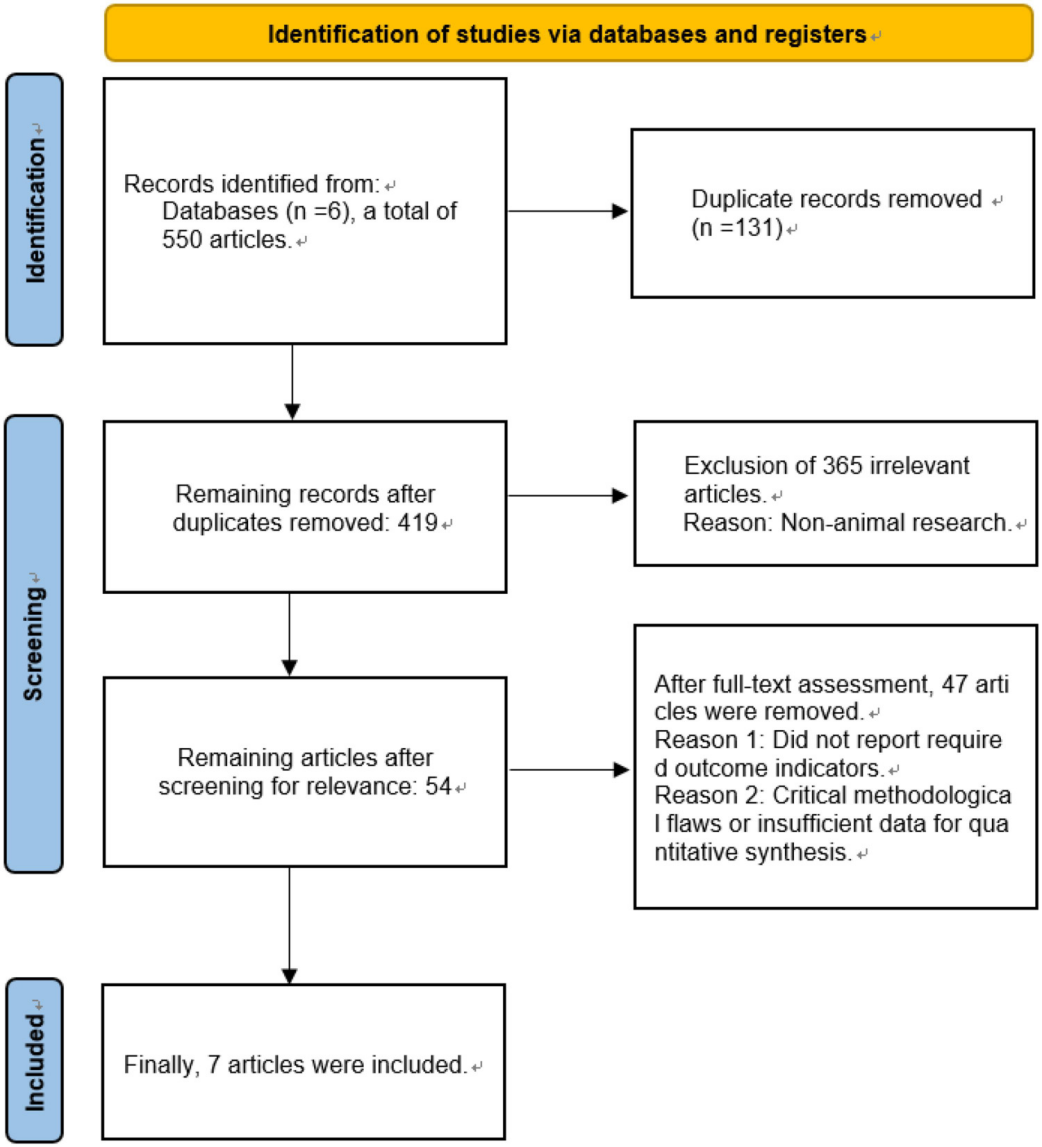
Flow chart of literature screening.

A two-phase filtration protocol was implemented for literature identification. Initially, two independent reviewers performed a preliminary screening based on the titles and abstracts of the retrieved articles. Subsequently, a detailed review of the full-text articles that passed the initial screening was conducted to finalize the selection of studies meeting the inclusion criteria. Conflicting assessments between reviewers were reconciled via structured discourse aiming for unanimous agreement, with third-party intervention mandated when unanimity proved unattainable. The entire screening process is clearly illustrated using a PRISMA flow diagram (Figure 2).

### 2.4 Data extraction

Two blinded investigators executed data abstraction via a protocol-driven extraction instrument, ensuring standardized collection. The extracted information included: basic study characteristics (e.g., first author, publication year, country, methodological framework, sample size); baseline characteristics of the animals (e.g., age, sex, disease diagnosis); detailed information on the interventions [including ESWT frequency, number of impulses, energy flux density, type of shockwave (radial or focal), and treatment protocol]; specifics of the control group; quantitative data for all prespecified outcome measures (e.g., mean, standard deviation, number of events); and reports of adverse events. For studies reporting on outcomes from multiple skeletal sites or with multiple intervention arms (e.g., different ESWT doses), data for each site or arm were extracted independently. To avoid unit-of-analysis errors, we handled these situations as follows: If a study featured multiple intervention arms (e.g., different ESWT parameters) compared to a single control group, we combined the intervention groups into a single group by calculating a pooled mean, standard deviation, and total sample size, following the methodology outlined in the Cochrane Handbook. For studies reporting outcomes from multiple skeletal sites, we selected data from a single, pre-specified site (prioritizing the femur, then the tibia) to ensure that each animal cohort was represented only once per outcome. For precision optimization, dual-researcher reconciliation preceded final validation, with the locked dataset undergoing independent authentication from an arbitration panel member.

### 2.5 Risk of bias assessment

Dual assessors independently appraised the methodological rigor and bias susceptibility of enrolled studies using Cochrane ROB 2.0 criteria. The risk of bias for the randomized controlled animal trials was evaluated using the SYRCLE's Risk of Bias tool, which covers domains including sequence generation, allocation concealment, baseline characteristics, blinding of investigators, blinding of animal care personnel, blinding of outcome assessors, incomplete outcome data, selective reporting, and other sources of bias. Each domain was judged as having a “low risk,” “high risk,” or “unclear risk” of bias. Employing the GRADE framework, we dynamically classified the certainty of evidence for critical outcomes across four tiers: high, moderate, low, or very low certainty.

**Figure 3:**
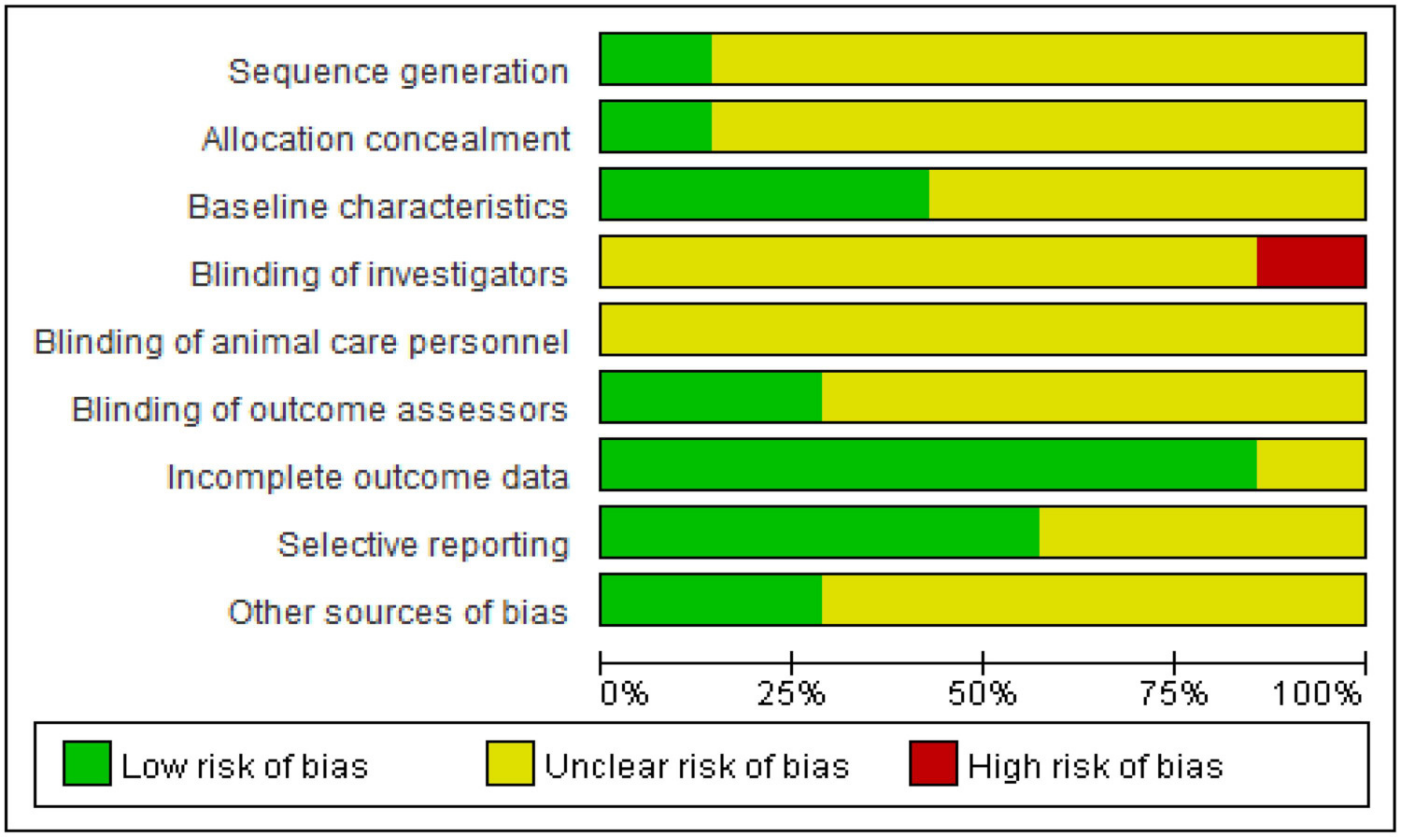
Outcomes of quality evaluation for selected literature.

**Figure 4:**
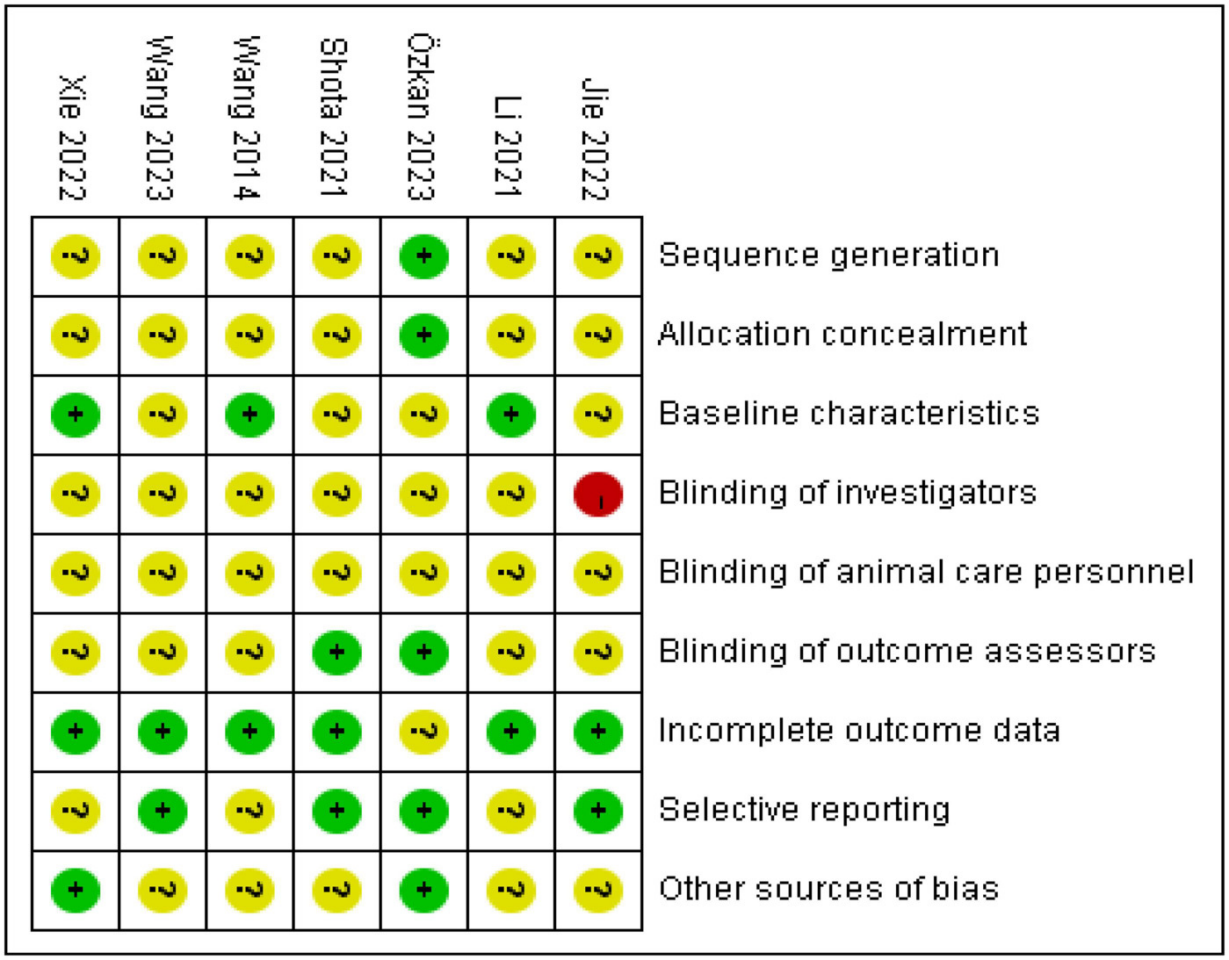
Summary map of included literature evaluation. “−” is high risk, “?” is unclear risk, and “+” is low risk.

Considering the inherent indirectness of extrapolating findings from animal models to humans, the initial quality of evidence for all outcomes was effectively considered “moderate”. This “moderate” starting point was then subject to further downgrading based on a systematic assessment of four key factors: risk of bias, inconsistency, indirectness, and imprecision, with a one-level downgrade for each serious issue identified. In this context, “inconsistency” refers to a very high and unexplained I² statistic, while “imprecision” refers to a very wide 95% confidence interval for the pooled effect size or failure to report it. The quality of evidence for outcome indicators is detailed in Table 1. Reviewer discrepancies were addressed through deliberative discussions, with an independent third researcher engaged for validation when warranted. Adherence to animal study reporting guidelines, such as ARRIVE, was also considered during the quality assessment process where applicable. The detailed assessments are presented in Figures 3, 4.

**Table 1:** Evaluation of evidence quality for outcome indicators.

Outcome Indicator	Number of Included Studies	Number of Animals	Pooled Effect Size (95% CI)	Quality of Evidence (GRADE)	Reasons for Downgrading
BMD	4	71	SMD: 2.12 (1.50, 2.73)	Moderate	Downgraded due to risk of bias ① and indirectness ②
BV/TV	3	35	SMD: 2.26 (0.20, 4.32)	Low	Downgraded due to risk of bias ①, indirectness ②, and small sample size
Tb.Sp	3	55	SMD: −1.39 (−2.64, −0.14)	Moderate	Downgraded due to risk of bias ① and indirectness ②
Tb.N	3	55	SMD: 3.51 (1.93, 5.10)	Low	Downgraded due to risk of bias ①, indirectness ②, and imprecision of results in some studies
Tb.Th	3	58	Md: 0.09 (0.04, 0.13)	Low	Downgraded Due To Risk Of Bias ①, Indirectness ②, And Some Studies Not Providing Confidence Intervals For Results

① The study did not report assigning concealment; ② Evidence comes from animal models of osteoporosis.

### 2.6 Outcome measures

The key outcome measures for this meta-analysis included bone mineral density (BMD) and a set of key trabecular microarchitectural parameters: bone volume fraction (BV/TV), trabecular separation (Tb. Sp), trabecular number (Tb. N), and trabecular thickness (Tb. Th). Both the densitometric (BMD) and microstructural outcomes were considered essential endpoints to provide a comprehensive assessment of ESWT's effects on bone health.

### 2.7 Statistical analysis

The research team performed all statistical analyses with RevMan 5.4 (Cochrane Collaboration's proprietary software platform). Given that all outcome measures of interest in this study were continuous variables, the choice of effect measure was based on the consistency of measurement methods and units. The Mean Difference (MD) with its 95% Confidence Interval (CI) was used for pooled analysis if the measurement methods and units were identical across studies; otherwise, the Standardized Mean Difference (SMD) with its 95% CI was employed. Inter-study heterogeneity was quantified using Cochran's *Q* test and the *I*² statistic. When the *P*-value of the *Q* test was ≥0.1 and *I*² < 50%, heterogeneity was considered low, and a fixed-effect model was used for data pooling. However, to conservatively account for potential unmeasured heterogeneity across studies, a random-effects model was applied for all outcomes, regardless of the *I*² value. Subgroup analyses were planned to explore the potential sources of heterogeneity. A sensitivity analysis, performed by sequentially removing one study at a time, was used to assess the robustness of the pooled results. Publication bias was evaluated by visual inspection of a funnel plot; if asymmetry was apparent, Egger's or Begg's test was used for further quantitative assessment. It is generally recommended that at least 10 studies are required for a reliable interpretation of funnel plot asymmetry and statistical tests for publication bias. The level of statistical significance was set at *P* < 0.05. All statistical analysis methods were reviewed by a biostatistics expert from Xi'an Physical Education University.

## 3 Results

### 3.1 Literature search and study inclusion

The preliminary literature retrieval yielded 550 pertinent records. After the removal of duplicates using EndNote software, 419 articles remained. In the primary screening phase, dual reviewers independently appraised bibliographic entries, discarding 365 records failing relevance thresholds. The 54 retained publications underwent full-text assessment, resulting in 7 RCT inclusions for meta-analysis based on protocol-defined criteria. Screening sequence is formally mapped in the PRISMA diagram (Figure 2). Reasons for full-text exclusion are detailed in Figure 2, primarily encompassing studies that did not report required outcome indicators or presented critical methodological flaws.

### 3.2 Characteristics of included studies

This meta-analysis ultimately included seven randomized controlled trials. Four experimental series employed ESWT to probe bone mineral density dynamics in established osteoporosis animal systems (14–17); three studies examined its effects on BV/TV (18–20); three studies on Tb. Sp (15, 18, 19), three studies on Tb. N (15, 18, 19), and three studies on Tb. Th (15, 19, 20). Comprehensive details of the included studies, covering authorship, publication details (year, country), methodology (design, sample size), animal baseline profiles, intervention and control conditions, and outcome assessments, are compiled in Tables 2, 3.

**Table 2:** Basic characteristics.

Study	Country	Article Type	N	Age (mean, SD)	Gender	Weight	Disease Type
Özkan (16) et al.	Turkey	RCT	13	8 months, not reported	F	2,750 g ± not reported	Induced osteoporotic rabbits
Wang et al. (17)	China	RCT	10	Not reported	F	150 g ± not reported	Induced osteoporotic rats
Jie et al. (20)	China	RCT	12	2.8 months, not reported	F	225 g ± not reported	Induced osteoporotic rats
Shota et al. (18)	Japan	RCT	9	5.5 months, not reported	F	300 g ± not reported	Induced osteoporotic rats
Li et al. (19)	China	RCT	14	8 months, not reported	F	Not reported	Induced osteoporotic rabbits
Wang et al. (14)	China	RCT	16	1.9 months, not reported	F	Not reported	Induced osteoporotic rats
Xie et al. (15)	China	RCT	32	Not reported	F	Not reported	Induced osteoporotic rats

**Table 3:** Study quality intervention treatment options.

Study	Treatment duration	Evaluation Metrics	Treatment Protocol	Types of Shock Waves	Parameters	Adverse Events
Özkan et al. (16)	3 weeks, once a week	①	Control group: Ovariectomy, no ESWT treatment; ESWT group: Received ESWT treatment	Radial-therapy	Pulses: 500, Frequency: 5 Hz, Energy Flux Density: 0.19 mJ/mm^2^	One death due to infection
Wang et al. (17)	8 weeks, once a week	①	Control group: Ovariectomy, received sham stimulation; ESWT group: Received ESWT treatment	Focused-therapy	Pulses: 1,500, Frequency: 5 Hz, Energy Flux Density: 0.35 mJ/mm^2^	Not reported
Jie et al. (20)	3 weeks, once a week	①②③④⑤	Control group: Ovariectomy, no ESWT treatment; ESWT group: Received ESWT treatment	Radial-therapy	Pulses: 2,000, Frequency: 4 Hz, Energy Flux Density: Not reported	Not reported
Shota et al. (18)	4 weeks, once a week	②③④⑤	Control group: Ovariectomy, no ESWT treatment; ESWT group: Received ESWT treatment	Radial-therapy	Pulses: 2,000, Frequency: 5 Hz, Energy Flux Density: 0.09 mJ/mm^2^	Not reported
Li et al. (19)	4 weeks, once every 3 days	②③④⑤	Control group: Ovariectomy, no ESWT treatment; ESWT group: Received ESWT treatment	Focused-therapy	Pulses: 2,000, Frequency: 4 Hz, Energy Flux Density: 0.12 mJ/mm^2^	Not reported
Wang et al. (14)	1 week, single treatment	①	Control group: Ovariectomy, no ESWT treatment; ESWT group: Received ESWT treatment	Focused-therapy	Pulses: 800, Frequency: Not reported, Energy Flux Density: 0.22 mJ/mm^2^	Not reported
Xie et al. (15)	8 Weeks, Once A Week	①③④⑤	Control Group: Ovariectomy, No ESWT Treatment; ESWT Group: Received ESWT Treatment	Unspecified	Pulses: 1,600, Frequency: 5 Hz, Energy Flux Density: 0.25 Mj/Mm^2^	Not Reported

① Bone mineral density (BMD); ② Bone volume fraction (BV/TV); ③ Trabecular separation (Tb. Sp); ④ Trabecular number (Tb. N); ⑤ Trabecular thickness (Tb.Th).

A notable characteristic of the included studies is the predominance of small-sample ovariectomized rat/rabbit models. While all animals were female, this aspect was not consistently highlighted across studies. Furthermore, there was considerable variability and inconsistent reporting of key study characteristics. Specifically, induction methods for osteoporosis, the exact skeletal sites targeted for ESWT, and the timing of intervention post-induction were not uniformly reported. Crucially, ESWT intervention parameters, including the specific modality (focused vs. radial), energy flux density (EFD), number of pulses, frequency, and anatomical target, were often inconsistently or incompletely described across studies. Moreover, no included study systematically reported any adverse events associated with ESWT treatment, indicating a significant gap in safety data.

### 3.3 Meta-analysis outcomes

Effects of ESWT on BMD, BV/TV, Tb. Sp, Tb. N, and Tb. Th in Animal Models of Osteoporosis.

#### 3.3.1 BMD

**Figure 5:**
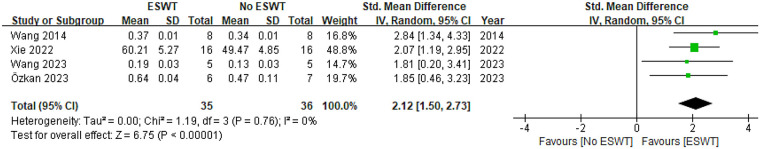
Pooled analysis of ESWT's impact on BMD within osteoporotic animal models.

Four studies reported on BMD (14–17), including a total of 35 animals receiving ESWT and 36 animals not receiving ESWT. Meta-analysis demonstrated a significant increase in BMD when comparing the ESWT group to the control (No ESWT) group. Under a random-effects model, the Standardized Mean Difference (SMD) was 2.12 with a 95% Confidence Interval (CI) of [1.50, 2.73]. As the entire 95% CI lies to the right of and does not include 0, this indicates a statistically significant positive effect of ESWT on BMD. The *Z*-test for the overall effect was 6.75 (*P* < 0.00001), further confirming this significance. The assessment of heterogeneity among the included studies showed no statistically significant heterogeneity (Tau² = 0.00, Chi² = 1.19, df = 3, *P* = 0.76; *I*² = 0%). An *I*^2^ value of 0% indicates extremely low inter-study variability and suggests high consistency across study results. Collectively, the four incorporated studies consistently demonstrated beneficial effects of ESWT on BMD. Furthermore, the 95% confidence intervals for all individual study estimates excluded the null value (0), confirming the statistical significance of each finding. See Figure 5 for details.

#### 3.3.2 BV/TV

**Figure 6:**
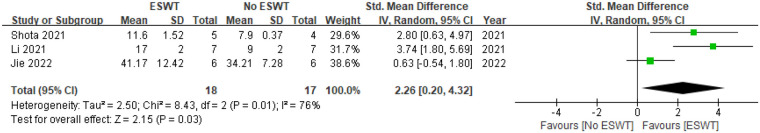
Pooled analysis of ESWT's impact on BV/TV within osteoporotic animal models.

Three studies reported on BV/TV (18–20), with a total of 18 animals in the ESWT group and 17 in the No ESWT group. The pooled analysis demonstrated a statistically significant increase in BV/TV between the two groups. The SMD under a random-effects model was 2.26 [95% CI (0.20, 4.32)]. The complete exclusion of the null value (0) by the entire 95% CI, positioned entirely to the right of zero, signifies a statistically significant benefit of ESWT on BV/TV. This significant effect was corroborated by the overall effect *Z*-test result (Z = 2.15, *P* = 0.03). The evaluation of between-study heterogeneity indicated substantial statistical differences (Tau² = 2.50, Chi² = 8.43, df = 2, *P* = 0.01; *I*² = 76%). The *I*^2^ value of 76% indicates high heterogeneity, suggesting that the results across studies may have considerable differences, which may warrant further investigation into the sources of this heterogeneity. Of the three included studies, two reported a significant positive effect of ESWT on bone, while one did not reach statistical significance. See Figure 6 for details.

#### 3.3.3 Tb. Sp

**Figure 7:**
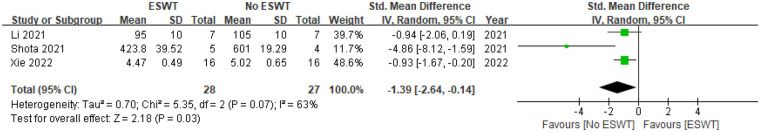
Pooled analysis of ESWT's impact on Tb. Sp within osteoporotic animal models.

Three studies reported on Tb. Sp (15, 18, 19), including 28 animals receiving ESWT and 27 not receiving ESWT. Meta-analysis revealed significantly different Tb. Sp values across the groups. The SMD under a random-effects model was −1.39 (95% CI [−2.64, −0.14]). As the entire 95% CI is to the left of and does not include 0, these results demonstrate a statistically significant therapeutic advantage of ESWT on Tb.Sp, indicating a decrease in trabecular separation (where negative values indicate ESWT group superiority). The *Z*-test for the overall effect was 2.18 (*P* = 0.03), confirming this significance. The assessment of heterogeneity indicated the presence of statistically significant and substantial variability across the studies (Tau² = 0.70, Chi² = 5.35, df = 2, *P* = 0.07; I² = 63%). According to our prespecified criterion (*P* < 0.10), this *P*-value confirms significant heterogeneity, a conclusion consistent with the high *I*² value. Of the three included studies, two reported a significant beneficial effect of ESWT, whereas one did not reach statistical significance. See Figure 7 for details.

#### 3.3.4 Tb. N

**Figure 8:**
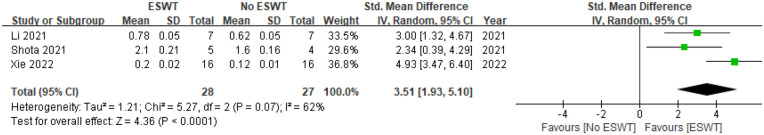
Pooled analysis of ESWT's impact on Tb. N within osteoporotic animal models.

Three studies reported on Tb. N (15, 18, 19), with a total of 28 animals in the ESWT group and 27 in the No ESWT group. The pooled analysis revealed a statistically significant increase in Tb. N between the groups. The SMD under a random-effects model was 3.51 [95% CI (1.93, 5.10)]. As the entire 95% CI is to the right of and does not include 0, this indicates a significant positive effect of ESWT on Tb.N. The *Z*-test for the overall effect was 4.36 (*P* < 0.0001), providing strong evidence for this significance. The assessment of heterogeneity revealed statistically significant and moderate-to-high variability among the studies (Tau² = 1.21, Chi² = 5.27, df = 2, *P* = 0.07; *I*² = 62%). In line with our prespecified methodology (*P* < 0.10), this *P*-value confirms that the inter-study heterogeneity is significant, a finding also supported by the I² value. Despite this heterogeneity, all three included studies individually reported a significant positive effect of ESWT on Tb. N. See Figure 8 for details.

#### 3.3.5 Tb. Th

**Figure 9:**
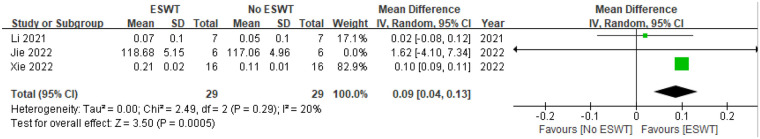
Pooled analysis of ESWT's impact on Tb. Th within osteoporotic animal models.

Three studies reported on Tb. Th (15, 19, 20), encompassing 29 animals in the ESWT group and 29 in the No ESWT group. The combined data analysis demonstrated a significant increase in Tb. Th between the groups. The MD under a random-effects model was 0.09 [95% CI (0.04, 0.13)]. Since the entire 95% CI is to the right of and does not include 0, this indicates a significant positive effect of ESWT on Tb. Th. The *Z*-test for the overall effect was 3.50 (*P* = 0.0005), further confirming this significance. Evaluation of between-study heterogeneity demonstrated the absence of significant statistical heterogeneity (Tau² = 0.00, Chi² = 2.49, df = 2, *P* = 0.29; *I*² = 20%). The *I*² value of 20% suggests low inter-study variability and good consistency across the results. In summary, the pooled analysis strongly supports a positive effect of ESWT on increasing trabecular thickness. Among the three individual studies, the findings from Xie 2022 were highly statistically significant, while the other two, though not reaching statistical significance, showed an effect direction consistent with the pooled result. See Figure 9 for details.

### 3.4 Publication bias

The likelihood of publication bias affecting results was estimated by visually evaluating funnel plot configurations for all outcome measures. For the outcomes of BMD and Tb. Th, the funnel plots displayed a relatively symmetrical distribution of study points, which were primarily concentrated in the top and central regions of the plot with no obvious gaps. This suggests the absence of significant publication bias for these indicators. However, for the outcomes of BV/TV, Tb. Sp, and Tb. N, the small number of included studies (fewer than 10 for each endpoint) limited the interpretability of the funnel plots. Although some asymmetry was observed, suggesting a potential risk of publication bias (e.g., studies with smaller or non-significant effects may not have been published), the limited number of studies precluded a definitive assessment. Due to the small number of included studies for most outcomes, formal statistical tests for publication bias (e.g., Egger's or Begg's test) were not performed, as their reliability is compromised with few studies.

### 3.5 Sensitivity analysis results

To determine result stability and identify heterogeneity origins, sensitivity analyses were performed on trabecular bone parameters (BV/TV, Tb. Sp, Tb. N).

BV/TV: The initial random-effects model for BV/TV indicated ESWT efficacy (SMD = 2.26, *P* = 0.03) despite significant heterogeneity (*I*² = 76%, 95% CI 0.20–4.32). Iterative sensitivity testing pinpointed Jie 2022 (20) as the key heterogeneity contributor. After exclusion, model homogeneity was restored (*I*² = 0%, Tau² = 0.00) with a recalculated SMD of 3.32 (95% CI 1.88–4.77, *P* < 0.00001). This 47.3% effect size increase and heterogeneity elimination suggests the robustness of ESWT's BV/TV enhancement and its biological plausibility (Table 4).

**Table 4:** Sensitivity analysis results of ESWT on BV/TV in osteoporotic animals.

Exclusion study	Heterogeneity test		Effect size		
Author	*I*^2^ value (%)	*P* value	SMD value	95% CI	*P* value
Shota et al. (18)	86%	0.007	2.09	(−0.96, 5.13)	0.18
Li et al. (19)	66%	0.08	1.52	(−0.57, 3.62)	0.16
Jie et al. (20)	0%	0.53	3.32	(1.88, 4.77)	<0.00001

Tb. Sp: Initial random-effects modeling for Tb.Sp demonstrated significant reduction with ESWT (SMD = −1.39, 95% CI −2.64 to −0.14; *P* = 0.03), though moderate heterogeneity was observed (*I*² = 63%). Iterative sensitivity testing pinpointed Shota 2021 (18) as the principal heterogeneity source. Post-exclusion analysis achieved complete model homogeneity (*I*² = 0%, Tau² = 0.00), concurrently enhancing both effect precision and magnitude: recalculated SMD improved to −0.93 (95% CI −1.55 to −0.32) with a 33.1% reduction in confidence interval width (2.50 → 1.23 units). Statistical significance strengthened 10-fold (*P* = 0.03 → 0.003), conclusively suggesting the robustness of ESWT's anti-separation effect on trabecular microstructure (Table 5).

**Table 5:** Sensitivity analysis results of ESWT on Tb. Sp in osteoporotic animals.

Exclusion study	Heterogeneity test		Effect size		
Author	*I*^2^ value (%)	*P* value	SMD value	95% CI	*P* value
Li et al. (19)	81%	0.02	−2.56	(−6.35, 1.23)	0.19
Shota et al. (18)	0%	0.99	−0.93	(−1.5, −0.32)	0.003
Xie et al. (15)	80%	0.03	−2.58	(−6.38, 1.21)	0.18

Tb. N: Initial random-effects synthesis for Tb.N revealed extreme ESWT efficacy (SMD = 3.51, 95% CI 1.93–5.10; *P* < 0.0001) despite moderate heterogeneity (*I*² = 62%). Iterative sensitivity testing identified Xie 2022 (15) as the dominant heterogeneity driver. Post-exclusion recalibration achieved complete model homogeneity (*I*^2^ = 0%, Tau² = 0.00), with a recalibrated SMD of 2.72 (95% CI 1.44–3.99) maintaining maximum statistical significance (*P* < 0.0001). The 22.5% effect size attenuation (3.51 → 2.72) accompanied by 19.6% confidence interval narrowing (3.17 → 2.55 units) demonstrates enhanced estimator precision, conclusively suggesting the robustness of ESWT's osteogenic capacity for trabecular number regeneration (Table 6).

**Table 6:** Sensitivity analysis results of ESWT on Tb. N in osteoporotic animals.

Exclusion study	Heterogeneity test		Effect size		
Author	*I*^2^ value (%)	*P* value	SMD value	95% CI	*P* value
Li et al. (19)	77%	0.04	3.72	(1.18, 6.26)	0.004
Shota et al. (18)	66%	0.09	4.01	(2.11, 5.90)	<0.0001
Xie et al. (15)	0%	0.62	2.72	(1.44, 3.99)	<0.0001

Ultimately, sensitivity analyses confirm that ESWT's beneficial impacts on enhancing BV/TV, decreasing Tb. Sp, and elevating Tb. N remain highly stable and reproducible, even after sequential exclusion of outlier studies driving initial heterogeneity.

## 4 Discussion

As the inaugural quantitative synthesis of evidence from seven randomized controlled trials in osteoporosis animal models, this systematic review and meta-analysis indicates that ESWT may have a beneficial effect on key bone microarchitectural parameters. The pooled results suggest that ESWT is associated with significant improvements in bone mineral density (BMD), bone volume fraction (BV/TV), trabecular number (Tb.N), and trabecular thickness (Tb.Th), as well as a reduction in trabecular separation (Tb.Sp). These preclinical findings provide an initial data foundation for further exploring ESWT as a potential non-invasive therapy for osteoporosis. However, it is crucial to interpret these results with caution, given the inherent limitations of preclinical data, including small sample sizes, heterogeneity across studies, and a lack of safety reporting.

While this meta-analysis offers initial support for ESWT, substantial heterogeneity was detected for BV/TV (*I*² = 76%), Tb.Sp (*I*² = 63%), and Tb.N (*I*² = 62%). Our qualitative analysis identified differing ESWT parameters (e.g., energy flux density, treatment duration, and frequency) as likely sources of this variability. For instance, the study by Jie 2022 (20), a key contributor to heterogeneity in the BV/TV analysis, had a shorter treatment duration and did not report the energy flux density. Similarly, variations in energy levels and treatment schedules in studies by Shota 2021 (18) and Xie 2022 (15) likely contributed to the heterogeneity in Tb.Sp and Tb.N analyses. This highlights the urgent need for standardized reporting in future preclinical studies, such as adherence to the ARRIVE guidelines, to facilitate more precise meta-analyses and determination of optimal treatment protocols.

The observed improvements in BMD and trabecular architecture are likely underpinned by several biological mechanisms, although the included studies in this meta-analysis focused primarily on morphometric outcomes rather than mechanistic pathways. Broader literature suggests ESWT influences bone metabolism through two main avenues: promoting bone formation and inhibiting bone resorption (21). With respect to bone formation (22), studies utilizing various *in vitro* and *in vivo* models (including some osteoporosis models) have shown that ESWT can enhance the transcriptional activity of osteogenic genes like Runx2, OCN, and ALP, and promote the differentiation of mesenchymal stem cells towards an osteoblastic lineage (18, 19, 23, 24). Furthermore, research often extrapolated from fracture healing models suggests ESWT upregulates crucial growth factors and signaling pathways, such as BMP−2 and Wnt/β-catenin, which are pivotal for osteogenesis (25–27). Regarding bone resorption, ESWT can upregulate the Nrf2/HO-1 pathway and has been reported to increase the OPG/RANKL ratio (28), thereby inhibiting osteoclastogenesis (29). It may also directly suppress osteoclast activity by reducing the expression of enzymes like cathepsin K and pro-inflammatory cytokines such as TNF-α (29–32). It is important to note, however, that the majority of these mechanistic insights are derived from studies not included in our meta-analysis, and direct evidence linking these pathways to the outcomes in the specific included papers is limited.

The translation of these preclinical findings to clinical practice for osteoporosis warrants careful consideration. A significant conceptual leap exists between the focal application of ESWT in animal limbs and its potential use for a systemic disease like osteoporosis in humans. Current successful human applications of ESWT, such as for fracture non-union and femoral head osteonecrosis, are for localized pathologies (33–35). Systemic osteoporosis, however, affects the entire skeleton, with the lumbar spine and proximal femur being clinically critical sites for fracture risk. A crucial unanswered question is the feasibility, efficacy, and safety of applying ESWT to these deep-seated and anatomically complex locations to achieve a clinically meaningful, systemic, or site-specific benefit. To date, the safety profile for such applications remains entirely unexplored. While some preliminary clinical studies on localized osteopenia show promise (36), they do not address the challenge of treating systemic osteoporosis.

This meta-analysis is subject to several important limitations. Primarily, evidence is exclusively from animal models, with inherent physiological differences [e.g., higher rodent bone turnover rates (37)] potentially limiting direct human translatability. Second, the small number of included studies (seven total, 3–4 per outcome) restricted statistical power, precluding robust subgroup or meta-regression analyses, and hindering definitive assessment of publication bias. The potential for selective reporting may also have led to an overestimation of the treatment effect. Third, most studies showed “unclear” or “high” risk of bias in key domains (e.g., allocation concealment, blinding), potentially compromising result validity. Fourth, ESWT safety data were largely undefined; only one study documented infection-related adverse events not linked to ESWT, with others reporting none, leaving potential risks unknown. Finally, ESWT modality varied (three radial, three focused, one unspecified). Despite the critical importance of comparing these distinct waveforms, a formal subgroup analysis was statistically unfeasible and unreliable due to the extremely limited number of studies within each modality subgroup.

## 5 Conclusion

In conclusion, this systematic review and meta-analysis of preclinical studies suggests that ESWT may improve bone density and microarchitecture in animal models of osteoporosis. However, due to significant limitations including the preclinical nature of the data, between-study heterogeneity, lack of safety reporting, and the major translational challenges of applying a focal therapy to a systemic disease, these findings should be considered preliminary. Rigorous, well-designed clinical trials are essential to determine the feasibility, safety, and efficacy of ESWT as a treatment for human osteoporosis, particularly at clinically relevant skeletal sites.
