# Supplementary figures and images for: The effects of extracorporeal shockwave therapy on bone mineral density and microarchitecture in animal models of osteoporosis: a systematic review and meta-analysis
Source: Front Rehabil Sci. 2026 Jan 12;6:1731044. doi: 10.3389/fresc.2025.1731044 (PMC12833336; doi:10.3389/fresc.2025.1731044)

1. 骨密度（BMD）


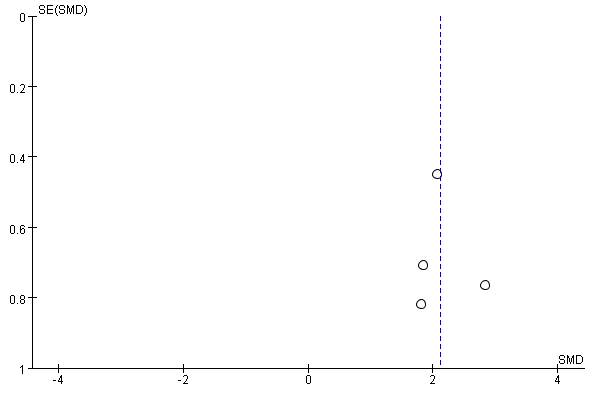


2. 骨体积分数（BV/TV）


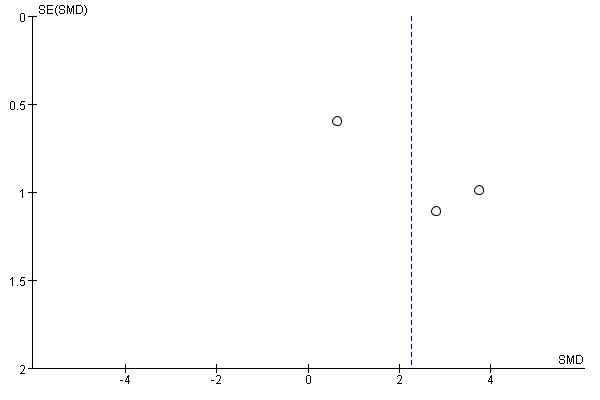


3. 骨小梁分离度（Tb，Sp）


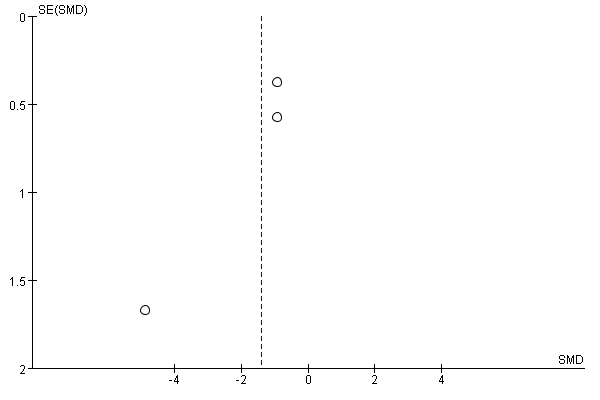


4. 骨小梁数量（Tb，N）


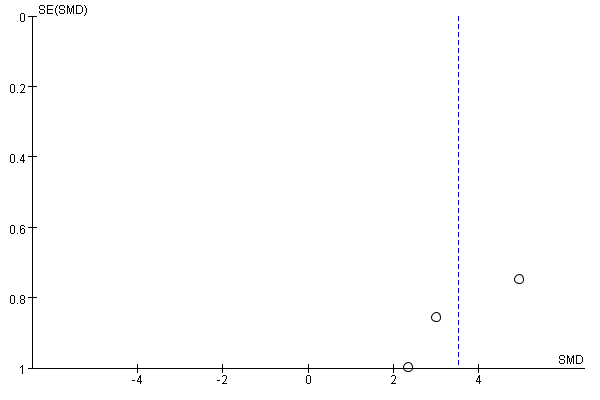


5. 骨小梁厚度（Tb，Th）


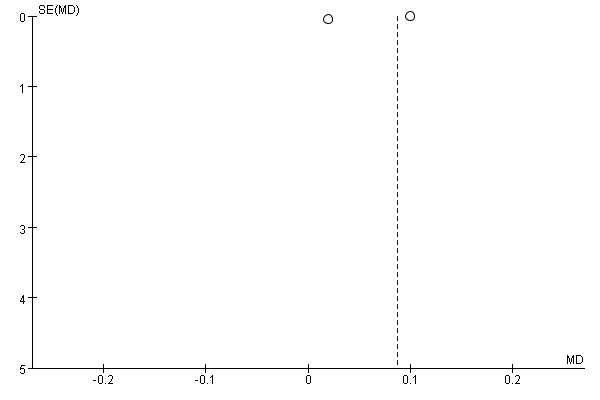

Supplement: Supplementary file 4 [file Table1.docx]
